# Untargeted metabolomics in *Anectocillus roxburghii* with habitat heterogeneity and the key abiotic factors affecting its active ingredients

**DOI:** 10.3389/fpls.2024.1368880

**Published:** 2024-03-08

**Authors:** Xinkai Lyu, Haixin Diao, Jiaxue Li, Zhixia Meng, Bing Li, Lisi Zhou, Shunxing Guo

**Affiliations:** ^1^ Institute of Medicinal Plant Development, Chinese Academy of Medical Sciences and Peking Union Medical College, Beijing, China; ^2^ State Key Laboratory of Basis and New Drug Development of Natural and Nuclear Drugs, Chinese Academy of Medical Sciences and Peking Union Medical College, Beijing, China

**Keywords:** untargeted metabolomics, *A. roxburghii*, habitat heterogeneity, keystone abiotic factors, flavonoids

## Abstract

**Introduction:**

*Anoectochilus roxburghii* is a rare, endangered herb with diverse pharmacological properties. Understanding the main metabolite types and characteristics of wild *A. roxburghii* is important for efficiently utilizing resources and examining quality according to origin.

**Methods:**

Samples were collected from the main production areas across five regions in Fujian Province, China. An untargeted metabolomics analysis was performed on the entire plants to explore their metabolic profiles. We utilized UPLC-MS/MS to specifically quantify eight targeted flavonoids in these samples. Subsequently, correlation analysis was conducted to investigate the relationships between the flavonoids content and both the biological characteristics and geographical features.

**Results:**

A comprehensive analysis identified a total of 3,170 differential metabolites, with terpenoids and flavonoids being the most prevalent classes. A region-specific metabolite analysis revealed that the Yongchun (YC) region showed the highest diversity of unique metabolites, including tangeretin and oleanolic acid. Conversely, the Youxi (YX) region was found to have the smallest number of unique metabolites, with only one distinct compound identified. Further investigation through KEGG pathway enrichment analysis highlighted a significant enrichment in pathways related to flavonoid biosynthesis. Further examination of the flavonoid category showed that flavonols were the most differentially abundant. We quantified eight specific flavonoids, finding that, on average, the YX region exhibited higher levels of these compounds. Correlation analysis highlighted a significant association between flavonoids and habitat, especially temperature and humidity.

**Discussion:**

Untargeted metabolomics via LC-MS was suitable for identifying region-specific metabolites and their influence via habitat heterogeneity. The results of this study serve as a new theoretical reference for unique markers exclusively present in a specific sample group.

## 1 Introduction

*A. roxburghii* (Wall.) Lindl, a rare, endangered herb from the Orchidaceae family. It has various pharmacological effects, such as lower blood glucose and blood lipids, protect the liver, and display anti-inflammatory and analgesic properties (Du et al., 2008). According to phytochemical studies, the ingredients in *A. roxburghii* responsible for the aforementioned biological activities are mainly flavonoids, terpenoids, phenols, organic acids, and alkaloids (Lyu et al., 2022). In China, *A. roxburghii* is mainly distributed in provinces south of the Yangtze River, such as Fujian, Zhejiang, Guangxi, and Yunnan (Ye et al., 2017). The morphological differences and biological characteristics of wild *A. roxburghii* vary among different regions. From the standpoint of contemporary analytical chemistry, the macroscopic distinctions evident in *A. roxburghii* arise from variances in their intrinsic chemical composition, which can be influenced by abiotic factors in different environments (Giraudo et al., 2019; Lu et al., 2021). For example, a higher light intensity enhances phenolic compound and organic acid production (Krzymińska et al., 2022), while temperature changes impact flavonoid (Goh et al., 2016) and terpenoid synthesis (Esposito et al., 2016). Therefore, revealing the chemical profiles and discriminating region-specific metabolites can provide a foundation for market standardization and cultivation technique optimization.

As of present, research concerning compounds within *A. roxburghii* remains notably limited, mainly focusing on about 100 compounds. There is a necessity for more extensive research methodologies to distinguish region-specific metabolites (He et al., 2005, 2006; Han et al., 2008; Wang et al., 2011; Chen et al., 2019; Wu et al., 2021). Untargeted metabolomics via LC-MS is effective for determining metabolic markers to assess the authenticity of biological identity (Zhou et al., 2019), pharmacological activity, and other factors (Zhong et al., 2022). One advantage of untargeted metabolomics is superior metabolite coverage, which detects as many metabolites as possible without any prior bias or knowledge (Mihailova et al., 2021). It can elucidate intricate metabolic processes in plants and reveal distinct plant growth patterns due to cultivar variances (Li et al., 2022), geographical settings (Long et al., 2023), and harvest periods (Shen et al., 2022). For instance, 22 unique metabolites were discerned in *Camellia drupifera* mature-seeds from Hainan and Liangguang by an untargeted metabolomic strategy based on UHPLC-QTOF-MS, highlighting their potential as region-specific markers (Xia et al., 2023). This study investigates the metabolic product differences between wild *A. roxburghii* from five different regions in Fujian Province and examines the influence of environmental factors on metabolic product accumulation. Fujian Province, a primary *A. roxburghii* production area, offers a diverse ecological environment that is an optimal habitat for growth and reproduction. Furthermore, Fujian has advanced artificial *A. roxburghii* cultivation systems with a relatively developed industry (Hong et al., 2015). The research also aims to provide references for screening region-specific metabolites and examining the influence of habitat heterogeneity.

## 2 Materials and methods

### 2.1 Samples

Wild *A. roxburghii* samples were collected from five typical geographical regions in China, including Nanping (NP, northern Fujian), Youxi (YX, eastern Fujian), Longyan (LY, western Fujian), Dehua (DH, southern Fujian), and Yongchun (YC, southern Fujian) in October 2020. The environmental parameters for these geographical locations were recorded, such as longitude, latitude, and altitude. The annual average temperature, average humidity, and average rainfall information was provided by the European Centre for Medium-Range Weather Forecasts. The samples were collected according to the principle of representativeness, and at least three different sampling points were selected for each region. The fresh *A. roxburghii* samples were randomly mixed after collection. The plants were packed, stored at 4 °C, and transported to the laboratory by air. Plants that were uniform in size, disease- and pest-free, and free from mechanical damage were selected for the experiments and washed with distilled water. All the samples were frozen with liquid nitrogen and stored at -80 °C for the subsequent experiments.

The plant heights (cm) and root lengths (cm) were measured with a ruler, while the fresh weight (g) values were obtained using an electronic balance. The number of leaves, stem nodes, and roots were counted. The leaf lengths (mm), the widths (mm) of the biggest leaves, and the maximum stem node lengths (mm) and widths (mm) were measured with a vernier caliper. The total root length (cm) was calculated. Duncan’s multiple range test was used to calculate the significant differences of each parameter across the five areas.

### 2.2 Metabolic analysis

A 50 mg sample was accurately weighed into a 1.5 mL Eppendorf tube, after which 800 µL of pre-cooled extract (methanol: water = 7:3, V:V) and 20 µL of internal standard were added and ground in a weaving grinder (50 Hz, 5 min) (JXFSTPRP, ShanghaiXinNing, China). Next, the sample was ultrasonicated in a 4 °C water bath for 30 min and placed in a refrigerator at -20 °C for 1 h, followed by centrifugation at 14,000 rpm for 15 min at 4 °C, after which 600 µL of the supernatant was collected. The supernatant was filtered using a 0.22 µm membrane, and the filtrate was transferred to a sample loading bottle for LC-MS analysis. Next, 20 µL of each sample was mixed to obtain QC samples to assess the repeatability and stability of the LC-MS analysis process.

A Hypersil GOLD aQ Dim column (1.9 µm 2.1*100 mm, Thermo Fisher Scientific, USA) was employed for chromatographic separation. Mobile phase A consisted of 0.1% formic acid in water, while mobile phase B comprised 0.1% formic acid in acetonitrile, and the column temperature was maintained at 40 °C. The gradient conditions included 5% B for 0.0-2.0 min, 5-95% B for 2.0-22.0 min, held constant at 95% B for 22.0-27.0 min, and washing with 95% B for 27.1-30 min. The flow rate was set at 0.3 mL/min, and the injection volume was 5 µL.

The Q Exactive instrument (Thermo Fisher Scientific, USA) was used for primary and secondary MS data acquisition. The positive ion mode scan range was set to 125-1500 m/z, while the negative ion mode range was 100-1500 m/z. The resolution was set to 70,000, and the automatic gain control (AGC) target for MS acquisition was set to 1e6, with a maximum ion injection time of 100 ms. For subsequent MSMS fragmentation, the top three precursors were selected with a maximum ion injection time of 50 ms and a resolution of 30,000, while the AGC was set to 2e5. The stepped normalized collision energy was set to 20 eV, 40 eV, and 60 eV. The electrospray ionization (ESI) parameters included a Sheath gas flow rate of 40, an Aux gas flow rate of 10, a spray voltage in positive-ion mode of 3.80 KV and 3.20 KV in negative-ion mode, a capillary temperature of 320 °C, and an Aux gas heater temperature of 350 °C.

### 2.3 Screening the differential metabolites of the various regions

A data matrix containing information, such as the metabolite peak areas and identification results, was obtained using the Compound Discoverer 3.2 (Thermo Fisher Scientific, USA) software and by analyzing the MS data according to the BMDB (BGI metabolome database), mzCloud, and ChemSpider online databases. SIMCA-P + 14.0 was performed for cluster analysis via PCA, PLS-DA, and OPLS-DA. The differential metabolites were identified via a statistically significant threshold of variable importance in projection (VIP) values obtained via OPLS-DA and Student’s t-test (*P-*value) of the raw data. The metabolites with fold changes ≥1.2 or ≤0.83 and q-value < 0.05 were considered statistically significant. The KEGG pathway database was used for taxonomic and functional annotation, while the top 20 pathways with *p*<0.05 values were selected for plotting. Standardized Z-scores >100 were selected for regional biomarker screening.

### 2.4 Screening the region-specific metabolites

A pairwise comparison of the metabolites of the five regions yielded 10 comparison groups. Metabolites with fold changes ≥1.2 or ≤0.83 and a q-value <0.05 were considered statistically significant. The differential metabolites of the 10 comparison groups were compared using an upset diagram created via MetaboAnalyst 5.0 (https://www.metaboanalyst.ca/MetaboAnalyst/faces/home.xhtml). YC-specific metabolites were common differential metabolites compared YC with other four regions (YC: NP, YC: DH, YC: LY, YC: YX), while absent in other regions pairwise comparison groups.

### 2.5 Quantitative analysis of 12 flavonoids via UPLC-MS/MS

Here, 20 mg of the powder was soaked in 3 mL of methanol, followed by ultrasonic extraction for 15 min at 100 kHz with 3 mL of methanol, 70% methanol, and water, respectively. The filtrate was mixed and passed through a 0.22-µm membrane. The mixed reference solution was prepared for Q1, Q3, limit of detection, precision, stability, repeatability, quantification, and standard curves. The mixed reference solution was continuously diluted until the signal-to-noise ratio reached 3, which was regarded as the limit of detection (LOD). Similarly, the signal-to-noise ratio reaching 10 was considered the limit of quantitation (LOQ). Stability tests involved standard sample solutions being subjected to room temperature for varying durations (0, 2, 4, 8, 12, 24, 36, and 48 hours), followed by injection, peak area recording, and RSD% calculation. Precision was evaluated by injecting the mixed reference solution diluent six times continuously, recording peak areas, and calculating RSD%. Reproducibility was assessed through the repetitive preparation of standard sample solutions (six times), injection for analysis, recording of peak areas, and RSD% calculation. The Shimadzu LC20A (Shimadzu, Japan) and 5500 QTRAP (AB SCIEX, USA) were used for primary and secondary MS data acquisition. A Waters ACQUITY UPLC BEH Shield RP18 (2.1 mm × 100 mm; 1.7 μm) (Waters, USA) was used for the 8-min separation process, using a mobile phase consisting of acetonitrile (A) and a 0.1% formic acid water solution (B), with gradient elution at a flow rate of 0.2 mL·min-1, a column temperature of 30 °C, and an injection volume of 2 μL. An ESI source was used for MS, while detection occurred in positive and negative ion modes simultaneously. Nitrogen was used at the spray and dry gas, with the curtain gas at 25 psi, a source temperature of 550 °C, a source injection voltage of +5500/−4500 V, and both the spray and auxiliary gas at 55 psi. **Supplementary Table 1** shows the quantitative and qualitative ions, declustering potential (DP), entrance potential (EP), collision energy (CE), and collision cell exit potential (CXP). Between-group comparisons were analyzed by Student’s t-test for unpaired data using SPSS 19.0, while differences at *p* ≤ 0.05 were considered significant. OmicStudio (https://www.omicstudio.cn/tool) was used for cluster heat map analysis, while Majorbio Cloud (www.majorbio.com) was employed for the Spearman correlation analyses of the active flavonoids accumulation, main climatic factors, and morphological attributes.

## 3 Results

### 3.1 Habitat climatic parameters and morphological attributes

**Figure 1:**
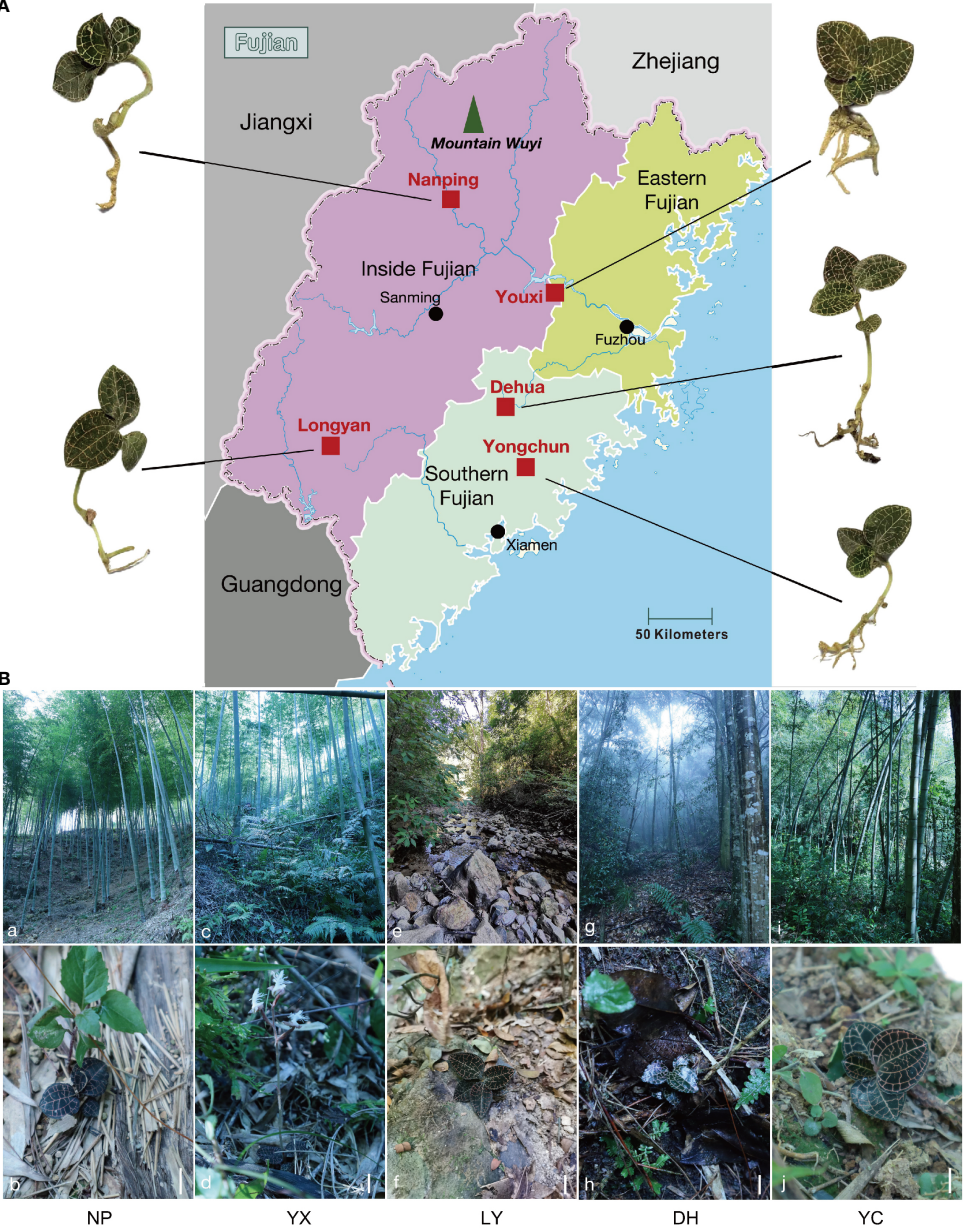
The sampling sites and growth environment of the wild *A. roxburghii*. The five wild *A. roxburghii* sampling sites **(A)**. The wild *A. roxburghii* growth environment **(B)**. a~b. The Nanping habitat. c~d. The Youxi habitat. e~f. The Longyan habitat. g~h. The Dehua habitat. i~j. The Yongchun habitat. The scales in the figures are all 3 cm. NP, Nanping; YX, Youxi; LY, Longyan; DH, Dehua; and YC, Yongchun.

**Figure 1A** shows the five sampling sites in this study, **Figure 1B** shows the growth environment of the five regions, while **Supplementary Table 2** presents the habitat climatic parameters. The *A. roxburghii* in NP, YX, and YC primarily grew in medium-density bamboo forests at different altitudes. NP has an altitude of 850 m, with spatial distribution on dry, aggregated soil, while YX is at an altitude of only 400 m, with more shrubs, resulting in higher density. The humidity in bamboo forests is low, with annual average values of 71.62% and 71.80% in YX and NP, respectively. Since YC is close to human settlements at an altitude of 650 m, it is significantly affected by human activities, showing an annual average humidity of 75%. The *A. roxburghii* in LY is mainly found scattered along streams and riverbeds at an altitude of only 350 m. The soil here is relatively humid, with notable sand particles. In DH, *A. roxburghii* is found in the light and humus layers of dense evergreen broad-leaved forests, where the altitude reaches 800 m, and the humidity and density are extremely high. The annual average humidity of LY and DH is approximately 77%.

A comparison between the morphological attributes (**Supplementary Figure 1**) showed that the various *A. roxburghii* indicators in broad-leaved forests in the DH region were generally higher than in other regions. The maximum leaf lengths, leaf widths, and root numbers in regions with higher humidity significantly exceeded those with lower humidity. Although the plant heights and stem numbers in DH and YX both substantially exceeded those in other regions, significant differences were evident between the environmental conditions of the two areas.

### 3.2 Overall metabolites analysis and multivariate analysis

**Figure 2:**
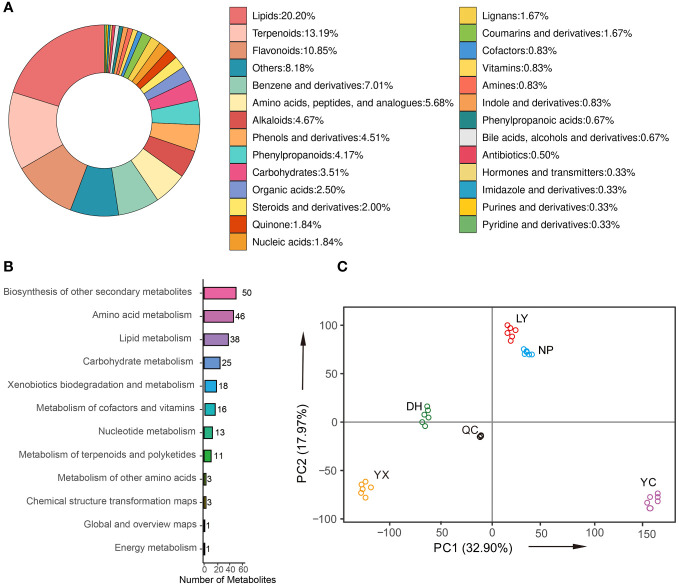
The classification of the identified metabolites **(A)**. The number of metabolites involved in the KEGG pathway **(B)**. PCA **(C)**. NP, Nanping; YX, Youx; LY, Longyan; DH, Dehua; and YC, Yongchun.

The typical base peak chromatogram (BPC) of QC samples in positive and negative ion modes are shown in **Supplementary Figure 2**, with good separation of individual ion peaks. A total of 4,990 metabolites (**Supplementary Table 3**) were identified and grouped into 17 classes (**Figure 2A**), including 20.20% lipids, 13.19% terpenoids, 10.85% flavonoids, 7.01% benzene and derivatives, 4.51% phenols and derivatives, 4.34% alkaloids, 4.17% phenylpropanoids, 4.01% amino acids, peptides, and analogs, 3.51% carbohydrates, and 2.5% organic acids. **Figure 2B** shows the number of identified metabolites involved in the KEGG pathway. The pathways displaying the highest metabolite abundance included secondary metabolites (50), amino acid metabolism (46), lipid metabolism (38), carbohydrate metabolism (25), xenobiotics biodegradation and metabolism (18), metabolism of cofactors and vitamins (16), etc. **Figure 2C** shows the PCA analysis of the QC and samples of the five regions. The QC samples were closely distributed, even overlapping near the coordinate axis origin, indicating that their metabolite content was similar. These findings indicated detection stability during the experiment and confirmed the reliability, accuracy, and reproducibility of the results. The different regional samples were distributed together without overlapping with other areas, indicating significant differences between the various *A. roxburghii* samples. LY and NP were relatively close, indicating that the metabolites of these two regions were similar.

### 3.3 The pairwise variation between the differential metabolites of the various regions

**Figure 3:**
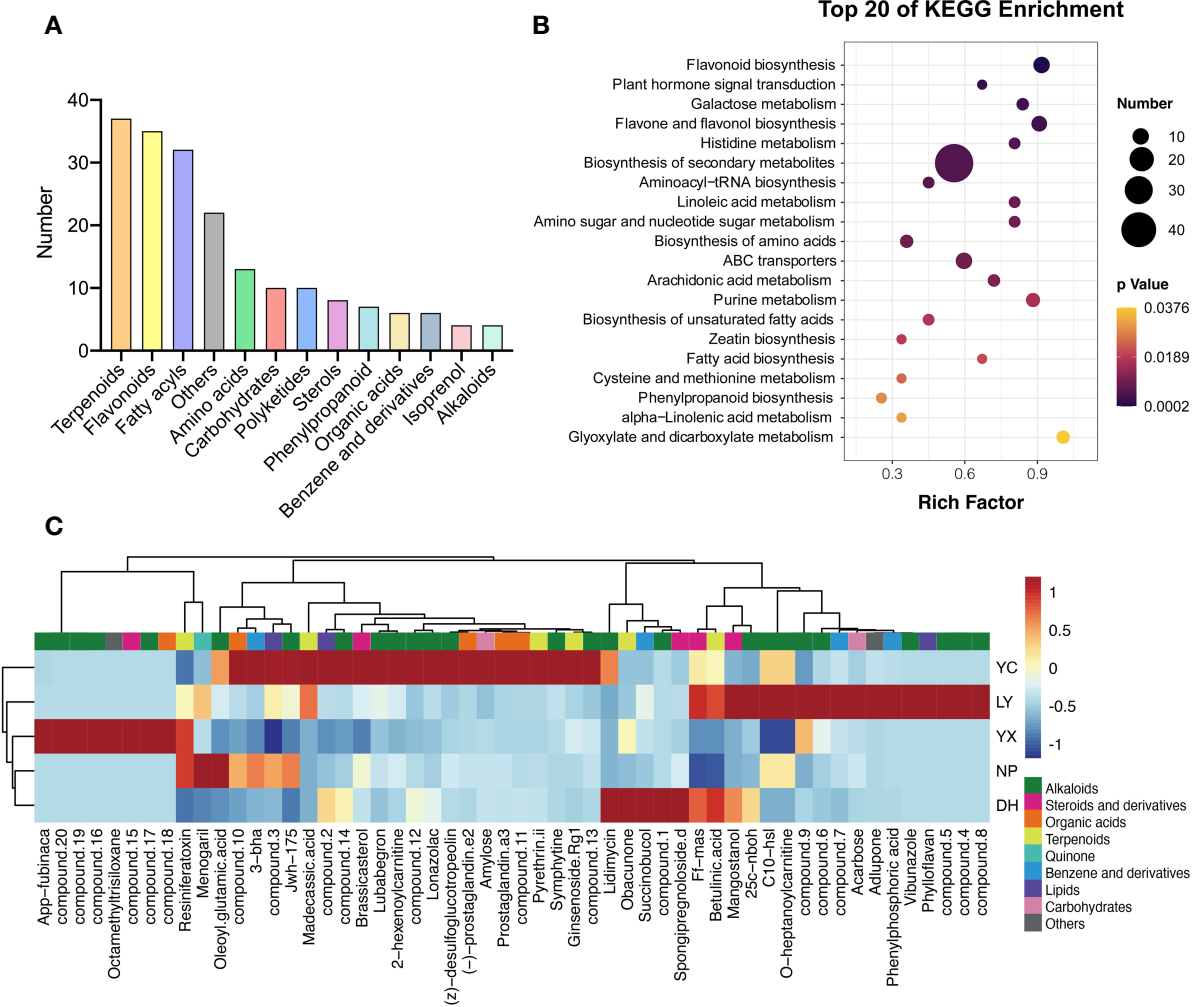
The classification of the differential metabolites **(A)**. The KEGG enrichment analysis of the differential metabolites **(B)**. The biomarker metabolites in each region **(C)**. NP, Nanping; YX, Youxi; LY, Longyan; DH, Dehua; and YC, Yongchun. The abbreviations for the compounds can be found in **Supplementary Table 5**.

A total of 3,170 differential metabolites were identified. They were divided into 4 levels. Level 1: substances are accurately identified using both the standard database and laboratory data, level 2: the structural formula matches that of substances in the standard database, level 3: the structural formula partially matches that of substances in the standard database, but further verification is required. And level 4: the accurate MS1 molecular weight matches that of substances in the database. The credibility decreases from Level 1 to Level 4 sequentially. We selected levels 1 and 2 with higher accuracy, yielding 376 differential metabolites, as shown in **Supplementary Table 4**. Of these, 194 were annotated, including 37 terpenoids, 35 flavonoids and flavonoid glycosides, 32 fatty acyls, 13 amino acids, 10 carbohydrates, nine organic acids, eight sterols, seven phenylpropanoids, six benzenes and their derivatives, and four isoprenols (**Figure 3A**). OPLS-DA was used to compare the differences between the regional groups (**Supplementary Figure 3**), showing excellent clustering without overlap. KEGG enrichment analysis indicated that the top-ranked pathways included flavonoid biosythesis, plant hormone signal transduction, galactose metabolism, flavone and flavonol biosythesis, histidine metabolism, biosythesis of secondary metabolites and so on (**Figure 3B**). These results indicated that flavonoids were the most dominant metabolites. Different metabolites with a Z-score>100 were screened in each group, leading to the observation of 54 metabolites. Metabolites exhibiting a Z-score greater than 100 were identified through screening within each group, revealing 54 metabolites. Consequently, a heatmap was constructed to depict the distribution of these metabolites. For enhanced visualization, regions manifesting a significantly elevated concentration of compounds were delineated, culminating in the generation of a novel regional biomarker map (**Figure 3C**). The results indicated that each region exhibited potentially unique marker metabolites, with alkaloids accounting for the largest proportion at a total of 25. YC presented the highest number of potential marker metabolites at 21, including lubabegron, symphytine, ginsenoside Rg1, prostaglandin a3, and 2-hexenoylcarnitine, while NP yielded the least at only three, including resiniferatoxin, menogaril, and oleoyl glutamic acid.

### 3.4 Region-specific metabolites

**Figure 4:**
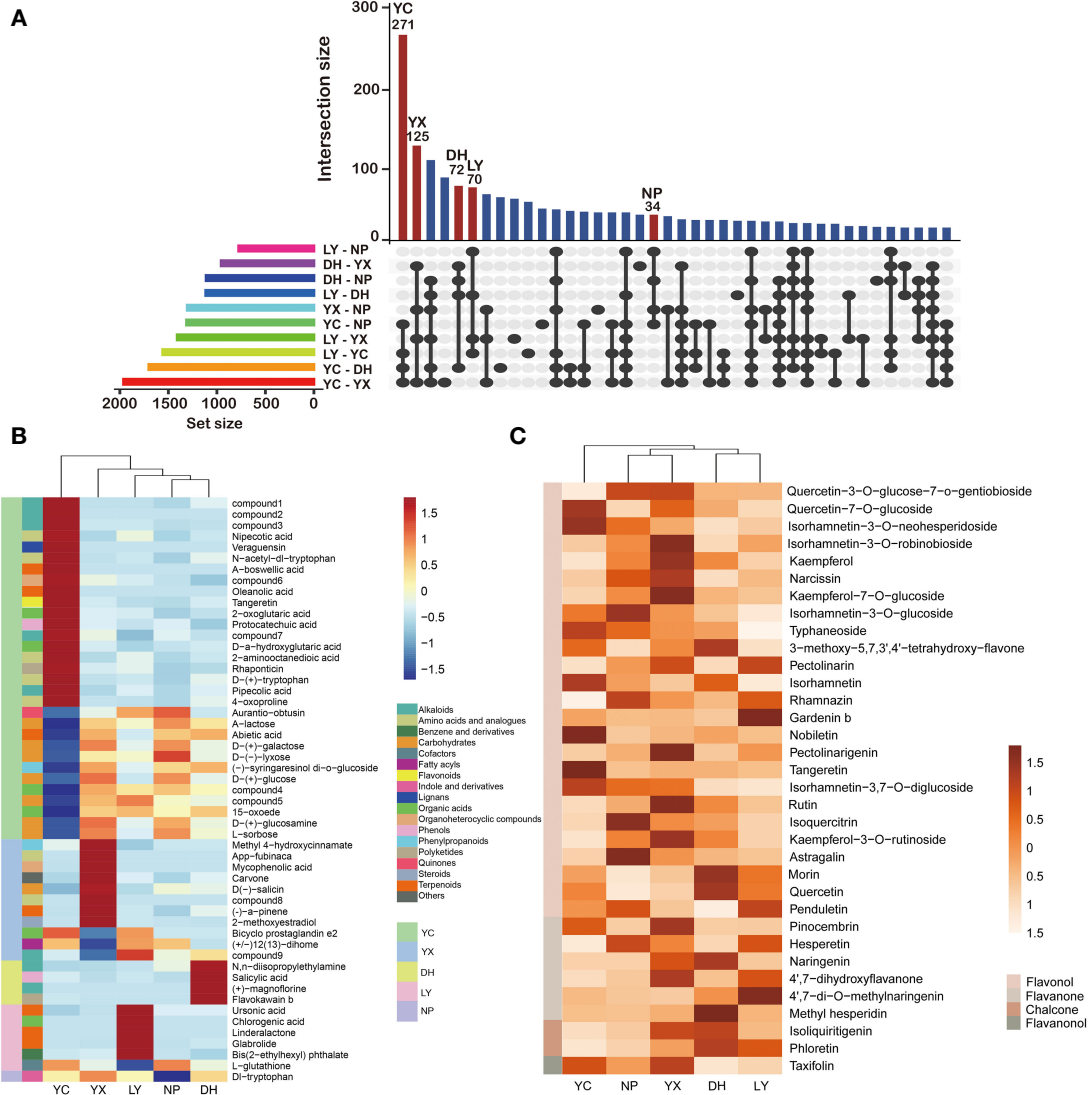
An upset diagram of the differential metabolites in the 10 comparison groups **(A)**. The region-specific metabolites in each region **(B)**. The regional characteristics of the flavonoids **(C)**. NP, Nanping; YX, Youxi; LY, Longyan; DH, Dehua; and YC, Yongchun. The abbreviations for the compounds can be found in **Supplementary Table 5**.

The differential metabolites of the five regions were compared in pairs to obtain an upset map of the 10 comparison groups (**Figure 4A**). Taking the first column as an example, it is a common intersection of the YC: NP, YC: DH, YC: LY, and YC: YX, but does not intersect with the other 6 comparison groups. Since these four groups are all compared with YC, it can be considered that the 271 differential metabolites in the first column are the YC-specific metabolites. According to this standard, we can find characteristic differential metabolites from the other four regions. Consequently, a total of 125 differential metabolites were identified as YX-specific matabolites, 72 for DH, 70 for LY, and 34 for NP. Only levels 1 and 2 were considered during the screening process to enhance the result accuracy, while a cluster heatmap was drawn to better distinguish the differences (**Figure 4B**). Here, 31 metabolites were selected as YC-specific, with 18 annotated as the most abundant, while NP yielded only one, namely dl-tryptophan. However, the differential metabolites were not significantly classified into a specific class of compounds.

### 3.5 Regional flavonoid characteristics

Preliminary findings indicated that flavonoids represented the main differential metabolites in the *A. roxburghii* from different regions. KEGG pathway enrichment analysis suggests that these differential metabolites are significantly enriched in the flavonoid biosynthesis and flavone and flavonol biosynthesis pathways. Further analysis annotated 48 flavonoids in the differentially accumulated metabolites, 34 of which were selected due to high accuracy (**Supplementary Table 6**). Flavonols were the most abundant, with 25 identified, including kaempferol, rutin, quercetin, naringenin, and isoquercitrin, while the six detected flavanones included hesperetin, naringenin, and 4’,7-dihydroxyflavone. Two chalcones, isoliquiritigenin and pinocembrin, were identified, along with one flavanonol, taxifolin. A cluster heatmap was generated (**Figure 4C**) to better illustrate the relative flavonoid content in the different regions, showing significant variations in the flavonoid composition. Each region displayed unique high-content flavonoids, such as rutin, kaempferol, and kaempferol-7-O-glucoside in YX, and quercetin-7-O-glucoside and hesperetin in YC. However, the flavanones, chalcones, and flavanonols were generally higher in YX and DH.

### 3.6 Quantitative analysis of 12 flavonoids via UPLC-MS/MS

**Figure 5:**
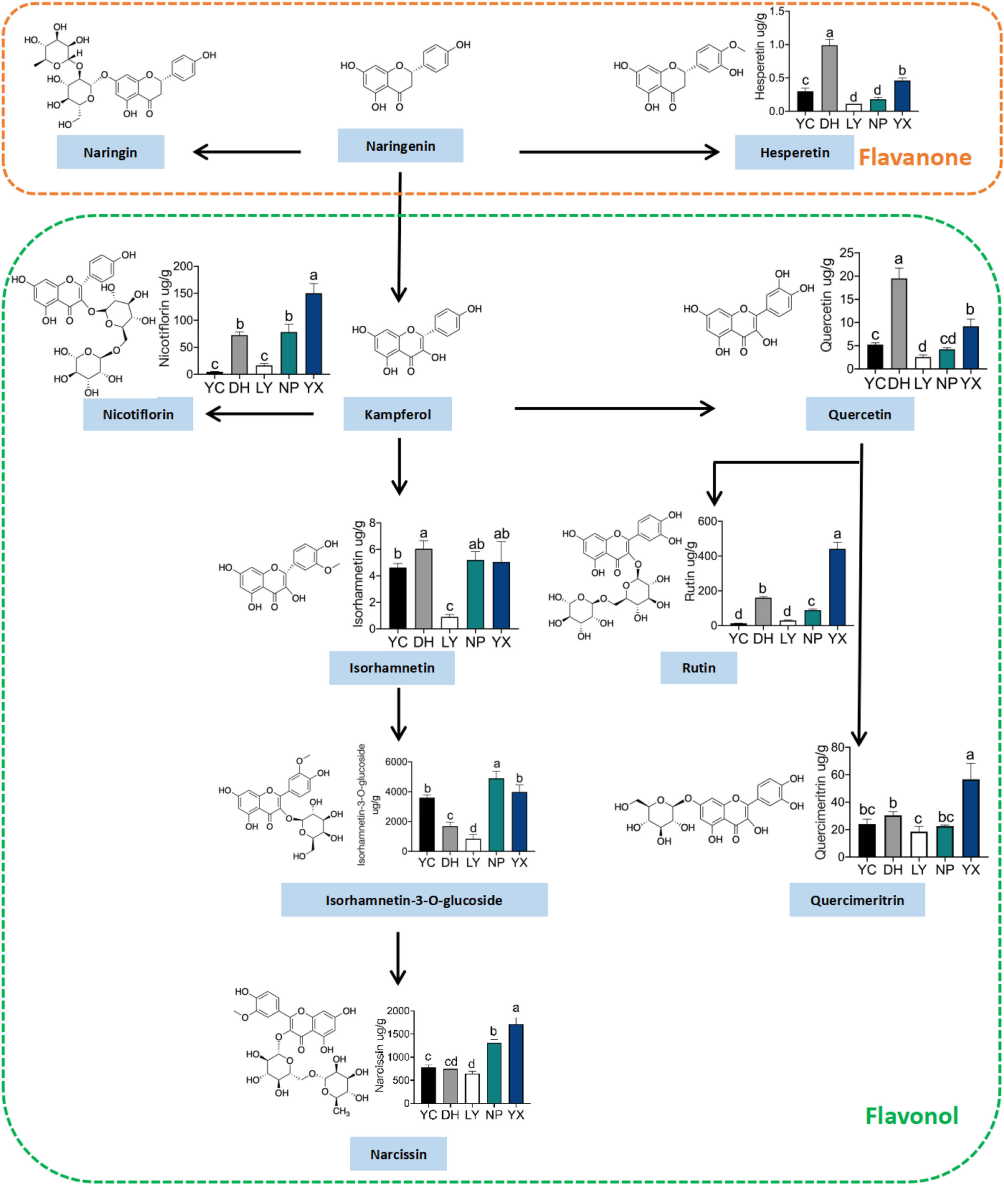
The flavonoid metabolite pathways and quantitative results. NP, Nanping; YX, Youxi; LY, Longyan; DH, Dehua; and YC, Yongchun. “a, b, c, d” represent significance (p < 0.05). When the letters on each bar chart are the same, they are not significant. Only when the letters are completely different, they are significant.

The levels of 12 different flavonoids in *A. roxburghii* from different regions were quantified to validate the accuracy of the metabolomics results. The metabolic pathways and quantitative analysis results are shown in **Figure 5**, primarily focusing on flavanone and flavonol metabolism. Flavanone metabolism included naringenin, hesperetin, and chrysin, while flavonol metabolism started with kaempferol and proceeds through one- and multi-step reactions to produce kaempferol-3-O-rutinoside, quercetin, isorhamnetin, isorhamnetin-3-O-glucoside, astragalin, rutin, and quercetin-7-O-glucoside. Additionally, the significantly different compound, chalcone, was quantified. **Supplementary Table 7** presents the method validation parameters for each compound. Eight flavonoids were ultimately quantified. Kaempferol, naringenin, pinocembrin, chrysin, and hesperetin were detected only at the detection limit and did not reach the quantification limit. The results indicated that the flavonoids in the same pathway showed consistent distribution trends across different regions. The only detected flavanone, hesperetin, displayed the lowest content of the eight flavonoids, with the highest level of 0.99 μg/g in DH, which was nine-fold lower than in LY. Of the flavonols, kaempferol-3-O-rutinoside was highest in YX, reaching 150.02 μg/g, which was 32.2-fold higher than in YC. The isorhamnetin, isorhamnetin-3-O-glucoside, and astragalin levels were higher in NP and YX and lowest in LY. Isorhamnetin exhibited extremely low levels in LY, at only 0.90 μg/g, while showing minimal variation in the other four regions. The isorhamnetin-3-O-glucoside level was the highest of the tested flavonoids, reaching 4902.29 μg/g in NP. The astragalin levels were substantially higher in YX than in the other four regions, reaching 1716.63 μg/g. Quercetin was highest in DH at 19.52 μg/g and lowest in LY at only 2.55 μg/g. The rutin and quercetin-7-O-glucoside levels were highest in YX, reaching 441.94 μg/g and 56.73 μg/g, respectively, which were 33.5- and 3.06-fold higher than in YC. In summary, YX displayed higher levels of the measured flavonoids, while LY generally exhibited lower levels, while the quantification and metabolomics results were consistent.

### 3.7 Correlation analysis between the active flavonoid accumulation and main climatic factors or morphological attributes

**Figure 6:**
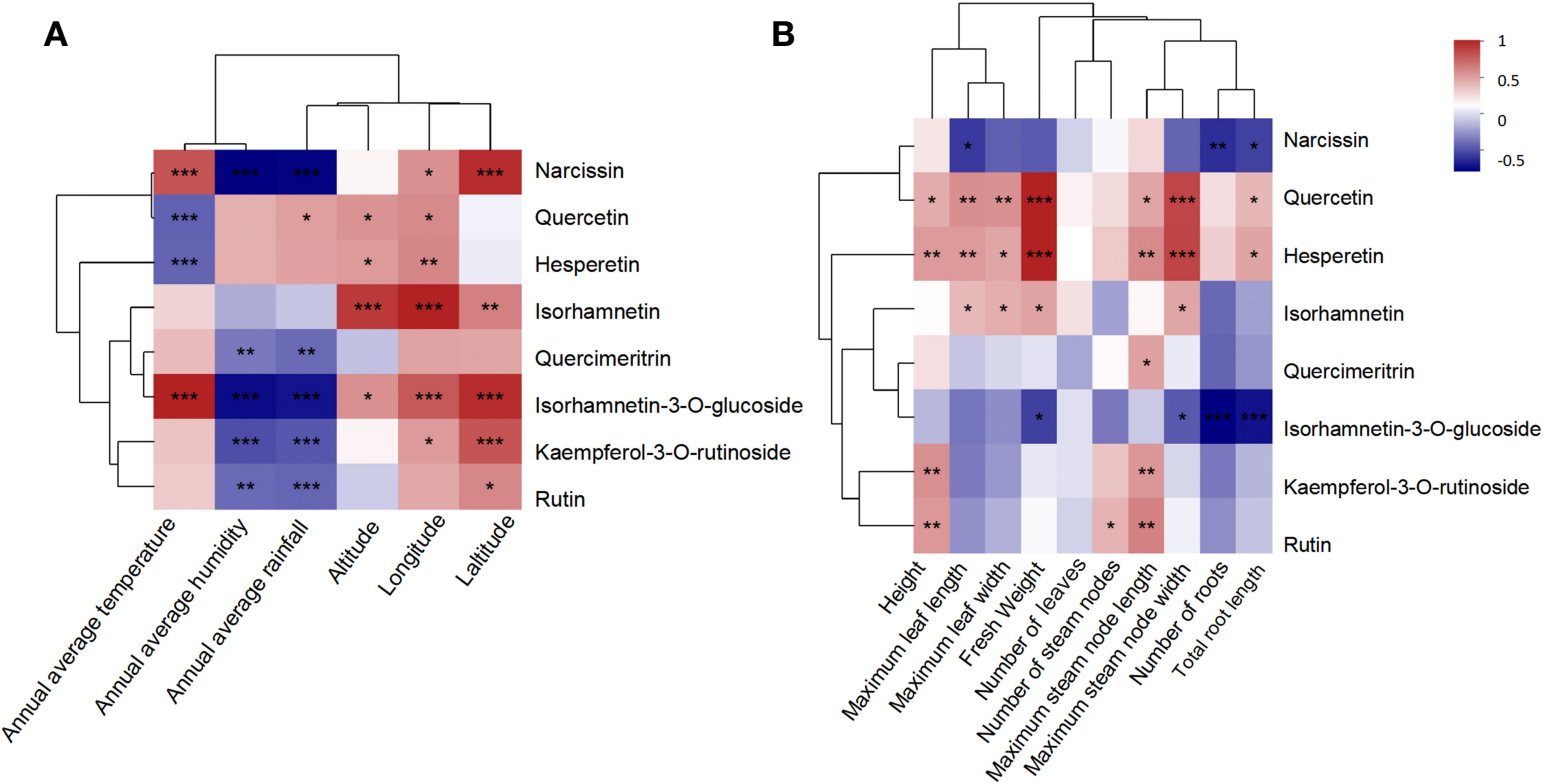
The correlation analysis between the quantitative flavonoids and geographical characteristics **(A)**. The correlation analysis between the quantitative flavonoids and biological characteristics **(B)**. NP, Nanping; YX, Youxi; LY, Longyan; DH, Dehua; and YC, Yongchun. The Pearson correlation coefficient (PCC) has a range of +1 (perfect correlation) to -1 (perfect but negative correlation), with 0 denoting the absence of a relationship. *: *P <*0.05; **P <0.01; ***P<0.001.

A correlation analysis between quantitative flavonoids and geographical and biological characteristics (**Figure 6**) showed that, except for quercetin and hesperin, six compounds exhibited consistent correlation with various factors, especially the high isorhamnetin-3-O-glucoside and narcissn levels, which were positively associated with temperature, longitude, and latitude, but negatively with humidity and rainfall. Furthermore, quercetin and hesperetin were positively correlated with all biological characteristics, indicating that their content increased in conjunction with vigorous *A. roxburghii* growth. However, narcissin and isorhamnetin-3-O-glucoside were negatively associated with most biological characteristics, particularly with the root abundance. Therefore, the content levels were lower in more robust plant roots. Isorhamnetin was more abundant in the *A. roxburghii* with lush leaves. Kaempferol-3-O-rutinoside and rutin were positively correlated with plant height, maximum stem length, and node number, suggesting that their content might increase as the *A. roxburghii* grew taller.

## 4 Discussion

### 4.1 The influence of habitat heterogeneity on region-specific metabolites

Currently, there are no comprehensive studies reporting on the metabolites of wild *A. roxburghii* analyzed based on metabolomics. However, some reports have compared the content of active ingredients in wild *A. roxburghii* from different regions using analytical chemistry techniques. Yan-bin Wu compared the flavonoid content in wild *A. roxburghii* from four regions of three provinces in China (Guangdong, Guangxi, Jiangxi) using HPLC-MS/MS. There were significant differences in flavonoid content among different regions. The content of hyperoside significantly higher than other flavonoids (Wu et al., 2020). These results consistent with ours. The content of quercetin, kaempferol, and isorhamnetin in these three provinces was significantly higher than that in Fujian. But the content of isorhamnetin-3-O-glucoside in Fujian was 15 to 333 times higher than that in these three provinces. These results suggested that these compounds can serve as biomarkers to differentiate it from different regions. Despite the overall subtropical climate of the five distinct geographical habitats in this study, variations in altitude and associated plant species resulted in slight differences in the soil environment and climate conditions between the regions, leading to significant variations in the *A. roxburghii* metabolites. Flavonoids in the *A. roxburghii* from the different regions showed substantial variation, suggesting that the geographic environment might influence flavonoid synthesis pathways, resulting in different flavonoid types and content across the regions (Ferreyra et al., 2021; Jia et al., 2023). Therefore, the diverse environmental conditions in different regions may activate specific metabolic pathways, producing distinct metabolites. *A. roxburghii* from eastern and southern Fujian, namely YC, YX, and DH, displayed a more varied characteristic metabolite profile than those from inner Fujian, LY, and NP. These differences can be attributed to variations in the semi-parasitic plant species, altitude, or soil in these regions (Huberty et al., 2020; Liu et al., 2023).

### 4.2 The effect of temperature changes on flavonoid accumulation

The antioxidant, anti-inflammatory, antibacterial, and antitumor properties flavonoids exhibit (Pourcel et al., 2007) play a crucial role in plant adaptability and defense mechanisms (Brunetti et al., 2013; Shah and Smith, 2020; Laoué et al., 2022; Zhuang et al., 2023). Therefore, the presence and content of flavonoids can reflect the physiological status and environmental adaptability of plants. Previous studies have revealed that heat stress can upregulate the genes related to the flavonoid pathway, increasing the quercetin, kaempferol, and isorhamnetin levels (Cui et al., 2023). Similarly, in tea, the genes associated with flavonoid glycosyltransferases are upregulated in high-temperature conditions, promoting flavonoid glycoside accumulation (Su et al., 2018). Low temperatures also affect flavonoid synthesis. In *Cryptomeria japonica*, exposure to cold stress can increase anthocyanin, flavonoid, and flavonol synthesis by upregulating FLS expression (Chalker-Scott, 1999). Correlation analysis of the quantitative flavonoids with temperature showed quercetin and hesperetin decreased in high-temperature conditions, the other six flavonoids were positively associated with temperature. Some of the result differs from previous studies. The formation of such differences is because that compound metabolism and biosynthesis are highly complex and influenced by various factors, including temperature, light, growth stage, and plant species. Therefore, specific regulatory mechanisms in different pathways and tissues may affect the observed correlations between flavonoids and temperature.

### 4.3 The influence of habitat heterogeneity on the key enzyme activity during flavonol biosynthesis

As some of the most abundant compounds in *A. roxburghii*, flavonoids are widely distributed in different regions, showing significant variation. Depending on the oxidation degree of the central three-carbon chain, the position of the B ring connection, and the presence of a cyclic structure in the three-carbon chain, primary natural flavonoids can be classified into fifteen types, including flavones, flavonols, flavonones, flavanones, anthocyanidins, flavan-3,4-diols, xanthones, chalcones, and bioflavonoids (Serafini et al., 2010; Shah et al., 2019). Analysis revealed significant variations between the flavonoids in different wild *A. roxburghii* regions, with flavonols dominating, followed by flavanones. In YX, the content of each flavonoid was relatively abundant, displaying the highest overall flavonoid content. The other *A. roxburghii* populations, primarily inhabiting bamboo forests, including NP and YC, showed relatively abundant overall flavonol and flavanonol levels. Contrarily, those growing under broadleaf canopies in DH and LY exhibited markedly different characteristics, with higher flavanone and chalcone levels. The structural analysis of the four flavonoid types indicated that flavanones and chalcones contained hydrogen (H) at position 3 of the C-ring, while flavonols and flavanonols possessed hydroxyl (OH) groups. Previous studies (Lukacin et al., 2003; Shen et al., 2006) have shown that the enzymes regulating the hydroxylation at position 3 in the flavonoid biosynthesis pathway primarily include flavanone 3-dioxygenase (1.14.11.9) and flavonol synthase (1.14.20.6). These enzymes catalyze early steps in the flavonoid biosynthesis pathway and require Fe2+ and ascorbate as cofactors (Xiong et al., 2016; Kumari et al., 2023). Therefore, it is hypothesized that the *A. roxburghii* in bamboo and broadleaf environments may be influenced by different iron ion levels or endophytic fungal types in their respective habitats. This can cause differences in flavanone 3-dioxygenase and flavonol synthase expression, affecting the early stages of flavonoid biosynthesis. Consequently, the *A. roxburghii* in these two habitats display flavonoid type variations, suggesting that bamboo or broadleaf components can be used as additives in culture media to selectively obtain specific types of flavonoids via artificial selection.

In conclusion, comprehensively examining the metabolic differences between the wild *A. roxburghii* from different regions can help understand its adaptation strategies and resource utilization. These findings hold important implications for the conservation, cultivation, and development of *A. roxburghii* resources. Future research can further investigate the metabolic pathways and potential medicinal value of *A. roxburghii* from specific regions to aid the comprehensive utilization and conservation of this valuable resource (Zhang et al., 2022).

## Data availability statement

The datasets presented in this study can be found in online repositories. The names of the repository/repositories and accession number(s) can be found below: MetaboLights, MTBLS8615.

## Author contributions

XL: Data curation, Formal analysis, Investigation, Methodology, Resources, Software, Validation, Visualization, Writing – original draft, Writing – review & editing. HD: Data curation, Formal analysis, Investigation, Methodology, Software, Writing – original draft. JL: Data curation, Formal analysis, Investigation, Methodology, Software, Writing – original draft. ZM: Conceptualization, Investigation, Methodology, Resources, Writing – review & editing. BL: Data curation, Investigation, Methodology, Resources, Software, Validation, Visualization, Writing – review & editing. LZ: Conceptualization, Data curation, Formal analysis, Investigation, Methodology, Project administration, Resources, Supervision, Validation, Visualization, Writing – original draft, Writing – review & editing. SG: Conceptualization, Data curation, Formal analysis, Funding acquisition, Investigation, Methodology, Project administration, Resources, Software, Supervision, Validation, Visualization, Writing – review & editing.

## Supplementary material

Supplementary Figure 1 The morphological attributes of A. roxburghii of five regions. Fresh weight **(A)**, height **(B)**, number of stem nodes **(C)**, maximum stem node length **(D)**, maximum stem node width **(E)**, number of blades **(F)**, maximum leaf length **(G)**, maximum leaf width **(H)**, number of roots **(I)**, total root length **(J)**. NP, Nanping; YX, Youxi; LY, Longyan; DH, Dehua; YC, Yongchun.

Supplementary Figure 2 Typical base peak chromatogram (BPC) of QC samples. BPC in negative ion mode **(A)**, BPC in positive ion mode **(B)**.

Supplementary Figure 3 The OPLS-DA analysis results of ten comparison groups. NP: Nanping, YX: Youxi, LY: Longyan, DH: Dehua, YC: Yongchun. DH vs NP **(A)**, DH vs YX **(B)**, DH vs LY **(C)**, LY vs NP **(D)**, LY vs YC **(E)**, LY vs YX **(F)**, DH vs YC **(G)**, NP vs YC **(H)**, YC vs YX **(I)**, NP vs YX **(J)**.

Supplementary Table 1 Mass spectrometry ion pairs and voltage conditions.

Supplementary Table 2 The habitat climatic parameters of five regions.

Supplementary Table 3 Compounds information identified by metabolomics.

Supplementary Table 4 376 Differential metabolites of five regions.

Supplementary Table 5 Summary of abbreviations for compounds in figure 3C and figure 4B.

Supplementary Table 6 34 flavonoids with high accuracy in the differentially accumulated metabolites.

Supplementary Table 7 Method validation parameters for 12 analytes.
