# Supplementary figures and images for: Untargeted metabolomics in Anectocillus roxburghii with habitat heterogeneity and the key abiotic factors affecting its active ingredients
Source: Front Plant Sci. 2024 Mar 8;15:1368880. doi: 10.3389/fpls.2024.1368880 (PMC10964796; doi:10.3389/fpls.2024.1368880)

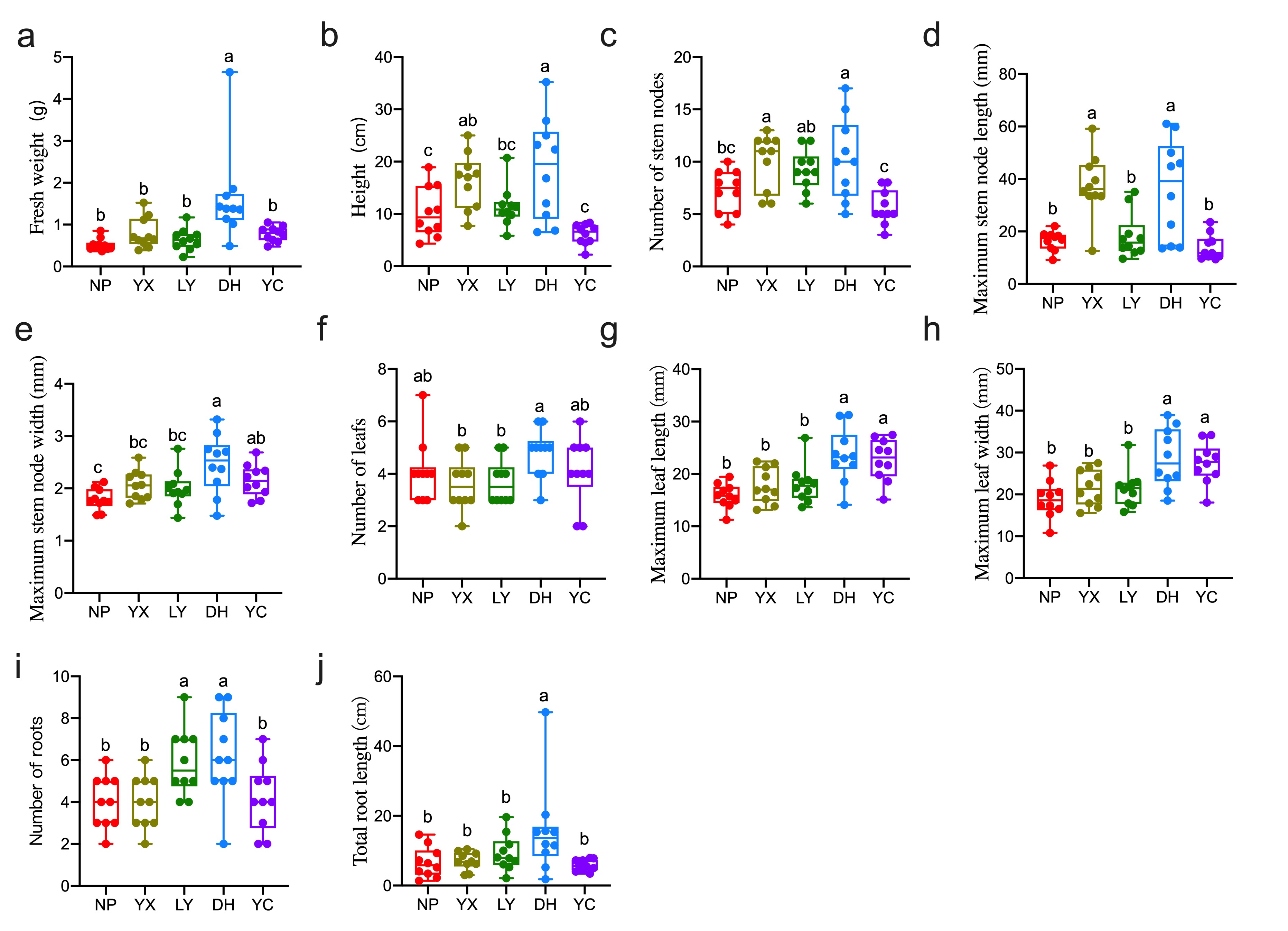

Supplement: Supplementary Figure 1 — The morphological attributes of A. roxburghii of five regions. Fresh weight (A), height (B), number of stem nodes (C), maximum stem node length (D), maximum stem node width (E), number of blades (F), maximum leaf length (G), maximum leaf width (H), number of roots (I), total root length (J). NP, Nanping; YX, Youxi; LY, Longyan; DH, Dehua; YC, Yongchun. [file Image_1.jpeg]

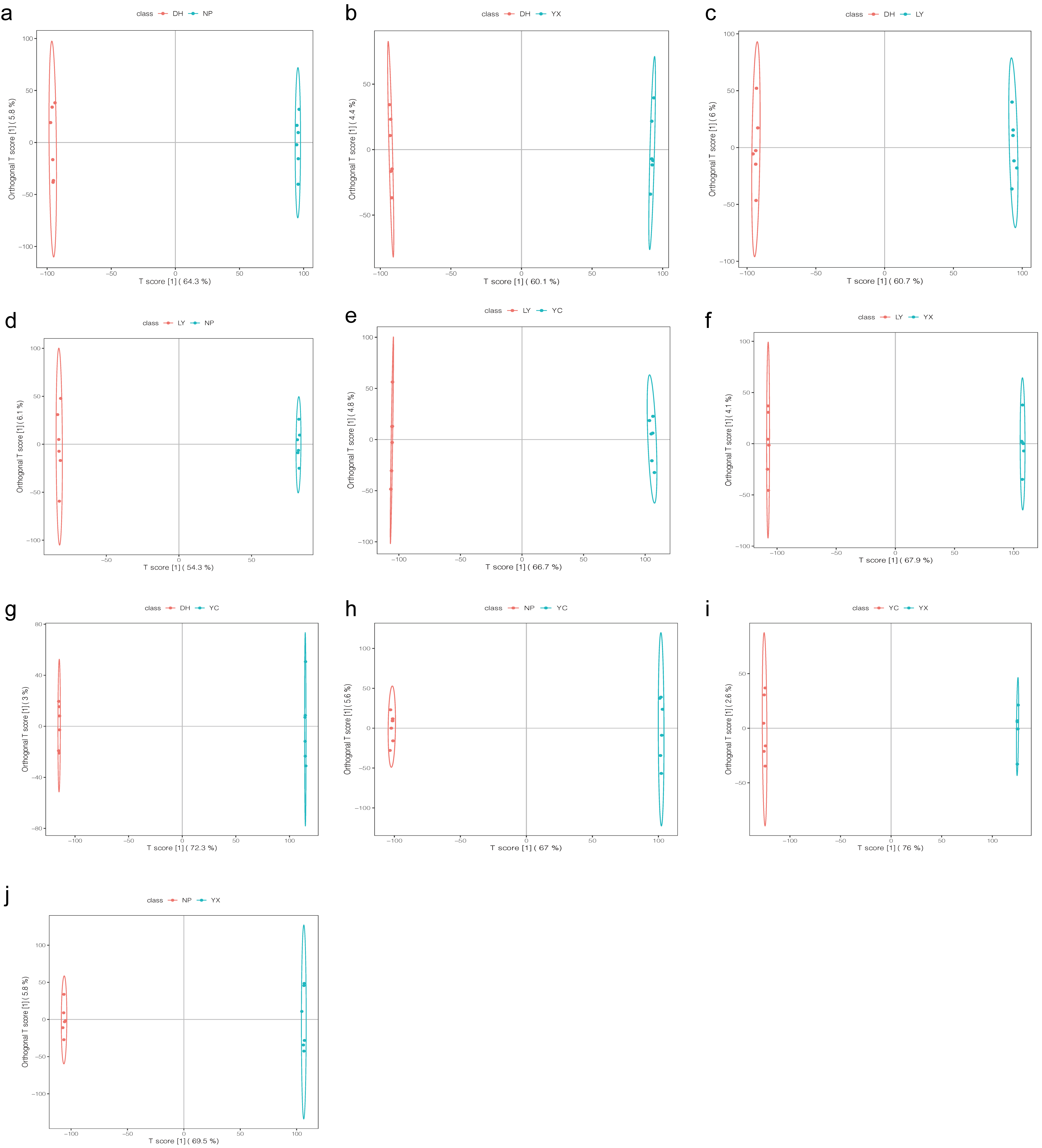

Supplement: Supplementary Figure 3 — The OPLS-DA analysis results of ten comparison groups. NP: Nanping, YX: Youxi, LY: Longyan, DH: Dehua, YC: Yongchun. DH vs NP (A), DH vs YX (B), DH vs LY (C), LY vs NP (D), LY vs YC (E), LY vs YX (F), DH vs YC (G), NP vs YC (H), YC vs YX (I), NP vs YX (J). [file Image_3.jpeg]
